# Cas1 and Cas2 From the Type II-C CRISPR-Cas System of *Riemerella anatipestifer* Are Required for Spacer Acquisition

**DOI:** 10.3389/fcimb.2018.00195

**Published:** 2018-06-12

**Authors:** Yang He, Mingshu Wang, Mafeng Liu, Li Huang, Chaoyue Liu, Xin Zhang, Haibo Yi, Anchun Cheng, Dekang Zhu, Qiao Yang, Ying Wu, Xinxin Zhao, Shun Chen, Renyong Jia, Shaqiu Zhang, Yunya Liu, Yanling Yu, Ling Zhang

**Affiliations:** ^1^Institute of Preventive Veterinary Medicine, Sichuan Agricultural University, Chengdu, China; ^2^Key Laboratory of Animal Diseases and Human Health of Sichuan Province, Chengdu, China; ^3^Avian Diseases Research Center, College of Veterinary Medicine, Sichuan Agricultural University, Chengdu, China; ^4^Department of Microbiology and Immunology, North Sichuan Medical College, Nanchong, China

**Keywords:** *Riemerella anatipestifer*, CRISPR-Cas, Cas1, Cas2, spacer acquisition

## Abstract

Clustered regularly interspaced short palindromic repeats (CRISPR) and CRISPR-associated (Cas) proteins provide acquired genetic immunity against the entry of mobile genetic elements (MGEs). The immune defense provided by various subtypes of the CRISPR-Cas system has been confirmed and is closely associated with the formation of immunological memory in CRISPR arrays, called CRISPR adaptation or spacer acquisition. However, whether type II-C CRISPR-Cas systems are also involved in spacer acquisition remains largely unknown. This study explores and provides some definitive evidence regarding spacer acquisition of the type II-C CRISPR-Cas system from *Riemerella anatipestifer* (RA) CH-2 (RA-CH-2). Firstly, introducing an exogenous plasmid into RA-CH-2 triggered spacer acquisition of RA CRISPR-Cas system, and the acquisition of new spacers led to plasmid instability in RA-CH-2. Furthermore, deletion of *cas1* or *cas*2 of RA-CH-2 abrogated spacer acquisition and subsequently stabilized the exogenous plasmid, suggesting that both Cas1 and Cas2 are required for spacer acquisition of RA-CH-2 CRISPR-Cas system, consistent with the reported role of Cas1 and Cas2 in type I-E and II-A systems. Finally, assays for studying Cas1 nuclease activity and the interaction of Cas1 with Cas2 contributed to a better understanding of the adaptation mechanism of RA CRISPR-Cas system. This is the first experimental identification of the naïve adaptation of type II-C CRISPR-Cas system.

## Introduction

*Riemerella anatipestifer* (RA) is a gram-negative bacterium that causes infection with high morbidity and mortality (Segers et al., 1993). RA infection occurs worldwide, primarily in domestic ducks, turkeys, and other birds, especially in 1–8-week-old ducklings (Ruiz and Sandhu, 2013). To date, more than 21 serotypes of RA have been isolated (Pathanasophon et al., 2002; Cheng et al., 2003). The complete genomic sequences of some RA strains have been submitted to the GenBank database in recent years (Wang X. et al., 2012; Wang et al., 2014; Zhu et al., 2016). Among these strains, *R. anatipestifer* CH-2 (RA-CH-2) is highly virulent and is one of the common serotype 2 strains in China (Wang et al., 2014). Research reports on RA are increasing and include studies on genomics (Wang et al., 2014; Liu J. et al., 2016; Song et al., 2016; Zhu et al., 2016), antibiotic resistance (Zhong et al., 2009, 2013; Luo et al., 2015, 2017; Huang et al., 2017; Zhang et al., 2017), energy metabolism (Liao et al., 2015, 2016; Liu et al., 2017a), physiology (Li et al., 2012; Yi et al., 2017), and virulence (Wang M. et al., 2017) of this organism and studies for method establishment (Wang X. P. et al., 2012; Gao et al., 2016; Liu M. et al., 2016; Liu et al., 2017b). However, studies on the physiology and pathogenic mechanism of RA are rare.

In many bacteria (~50%) and most archaea (~87%), a novel immune system comprised of clustered regularly interspaced short palindromic repeats (CRISPR) and the CRISPR-associated (Cas) protein has been identified, and this system can provide acquired genetic immunity against the entry of mobile genetic elements (MGEs), such as viruses (phage) and plasmids (Makarova et al., 2015). The immune system relies on the Cas protein (the effector module) to cleave specific sequences of MGEs, guided by CRISPR RNA (crRNA) (Barrangou, 2013). According to the current classification based on multiple criteria, CRISPR-Cas systems are divided into two classes that comprise six types and 19 subtypes (Koonin et al., 2017). Among these systems, class II CRISPR-Cas system is the simplest and contains a single effector protein. Among the class II systems, type II CRISPR-Cas systems are the most common and best understood, especially type II effectors (Shmakov et al., 2017). The subtype II-C CRISPR-Cas system comprises ~41% of type II systems, contains a mini *cas* operon (Shmakov et al., 2017), uses Cas9 protein as an effector and lacks Csn2 protein (Koonin et al., 2017). Systems with only three *ca*s genes (*cas1, cas2*, and *cas9*) in the operon were originally defined as type II-C CRISPR-Cas systems (Chylinski et al., 2014). Burstein et al. reported a type II-C system variant with *cas4* and considered it to be a fusion of type II-C and II-B systems (Burstein et al., 2017). The numerous and varied type II-C CRISPR-Cas systems remain to be explored.

Despite the extreme diversity in the organization of CRISPR-Cas loci, the CRISPR/Cas-mediated immune defense includes three phases: adaptation (spacer acquisition), expression (crRNA biogenesis), and interference (cleavage of target DNA or RNA) (Marraffini, 2015). It is generally known that the nearly universal Cas1 and Cas2 function in the early stage of the diverse CRISPR-Cas systems as adaptation proteins (Yosef et al., 2012; Arslan et al., 2014; Heler et al., 2015; Nuñez et al., 2015; Wei et al., 2015b; Fagerlund et al., 2017; Xiao et al., 2017). In addition to the functions of Cas1 and Cas2, various requirements for spacer acquisition must be met in the diverse CRISPR-Cas systems. For example, Cas adaptation proteins recognize only protospacers (the sequences from which spacers are derived) that are closest to the protospacer adjacent motif (PAM) to select and insert into the CRISPR array (Mojica et al., 2009; Wang et al., 2015; Leenay and Beisel, 2017). In addition, the leader sequence (generally A/T-rich) (Yosef et al., 2012; Wei et al., 2015a; McGinn and Marraffini, 2016), CRISPR repeat (Yosef et al., 2012; Wei et al., 2015a), host factor (Nuñez et al., 2016; Yoganand et al., 2017), and/or other Cas proteins (Heler et al., 2015; Wei et al., 2015b) are also required for spacer acquisition in various CRISPR-Cas systems.

**Figure 1:**
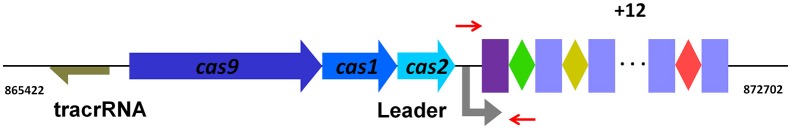
Organization of the type II-C CRISPR-Cas system from RA-CH-2. The CRISPR1 locus from RA-CH-2 comprises three *cas* genes (*cas9, cas1*, and *cas2*), 16 repeats (purple or blue rectangles, 47 bp long) and 15 spacers (colored diamonds, 30 bp long). The tracrRNA (trans-activating crRNA, brown arrow) containing the region with complementarity to the repeats is located upstream of cas9. Between *cas2* and the first repeat (purple rectangle), the A/T-rich region is the presumed leader sequence. The primers (short red arrows) were used for PCR amplification to detect CRISPR1 expansion.

Our previous study revealed that RA encoded the type II-C CRISPR-Cas system harboring a Cas9 effector (Zhu et al., 2016). Two CRISPR arrays were found in RA-CH-2. In addition, CRISPR1 was shown to be flanked on one side by *cas* genes, while CRISPR2 was designated an orphan. The organization of CRISPR1-Cas locus of RA-CH-2 (Figure 1) demonstrates that RA-CH-2 CRISPR-Cas system is a type II-C system with the Cas9 effector protein and lacking both Cas4 and Csn2. Only 2 spacers (2/32) were homologous with MGEs (phage sequences) and were located in RA-CH-2 CRISPR1 array. Putative protospacers were present in the genomes of *Riemerella* phage RAP44 (NC_019490.1, 100% similarity) and *Enterobacteria* phage phi92 (NC_023693.1, 87% similarity). However, no experimental evidence has been provided regarding RA CRISPR-Cas system. In this study, assays of spacer acquisition and plasmid loss were carried out in RA-CH-2 strain. In addition, the nuclease activity of RA Cas1 and the interaction of Cas1 with Cas2 were investigated. The above assays contribute to a better understanding of the adaptation of RA CRISPR-Cas system.

## Materials and methods

### Bacterial strains, plasmids, and culture conditions

All strains and plasmids used in this study are listed in Table 1, and primers are listed in Table 2. RA cells were grown at 37°C in tryptic soy broth (TSB) or on tryptic soy agar (TSA) (Difco Laboratories, USA) supplemented with 5% calf serum (Biologos, USA) in a 5% CO_2_ atmosphere. *Escherichia coli* cells were cultured at 37°C in Luria-Bertani (LB) broth or on LB agar (LBA) plates. Isopropyl β-D-1-thiogalactopyranoside (0.5 mM; IPTG; Sigma, USA) was used to induce promoter expression. When necessary, antibiotics were added at the following concentrations: ampicillin (Amp) at 100 μg/ml and kanamycin (Kan) at 50 μg/ml for *E. coli* and spectinomycin (Spc) at 50 μg/ml and cefoxitin (Cfx) at 1 μg/ml for RA.

**Table 1:** Bacterial strains and plasmids used in this study.

	**Genotype**	**Source or reference**
***E. coli*** **STRAIN**		
BL21(DE3)	Expressing host cell	Laboratory collection
BL21(DE3) pET30a::*cas1*	*E. coli* BL21(DE3) carrying pET30a::*cas1*, Kan^R^	This study
BL21(DE3) pET30a::*cas1^*E*149*A*^*	*E. coli* BL21(DE3) carrying pET30a::*cas1^*E*149*A*^*, Kan^R^	This study
BL21(DE3) pET30a::*cas1^*H*206*A*^*	*E. coli* BL21(DE3) carrying pET30a::*cas1^*H*206*A*^*, Kan^R^	This study
BL21(DE3) pET30a::*cas1^*E*221*A*^*	*E. coli* BL21(DE3) carrying pET30a::*cas1^*E*221*A*^*, Kan^R^	This study
BL21(DE3) pET30a::*cas1-cas2*	*E. coli* BL21(DE3) carrying pET30a::*cas1-cas2*, Kan^R^	This study
DH5a pGEM::CRISPR	*E. coli* DH5a carrying pGEM::CRISPR, Amp^R^	This study
***R. anatipestifer*** **STRAIN**		
RA-CH-2	Serotype 2, Cfx^S^, Spc^S^	Laboratory collection
RA-CH-2 pLMF03	RA-CH-2 carrying pLMF03, Cfx^R^, Spc^S^	This study
RA-CH-2 Δ*cas1*	*cas1* deletion mutant of *R. anatipestifer* CH-2 strain, Cfx^S^, Spc^R^	This study
RA-CH-2 Δ*cas2*	*cas2* deletion mutant of *R. anatipestifer* CH-2 strain, Cfx^S^, Spc^R^	This study
RA-CH-2 Δ*cas1-cas2*	*cas1-cas2* deletion mutant of *R. anatipestifer* CH-2 strain, Cfx^S^, Spc^R^	This study
RA-CH-2 Δ*cas1* pLMF03	RA-CH-2 Δ*cas1* carrying pLMF03, Cfx^R^, Spc^R^	This study
RA-CH-2 Δ*cas2* pLMF03	RA-CH-2 Δ*cas2* carrying pLMF03, Cfx^R^, Spc^R^	This study
RA-CH-2 Δ*cas1-cas2* pLMF03	RA-CH-2 Δ*cas1-cas2* carrying pLMF03, Cfx^R^, Spc^R^	This study
**PLASMID**		
pET30a	pBR322 lacZ, IPTG-inducible promoter, Kan^R^	Laboratory collection
pET30a::*cas1*	pET30a carrying *cas1* from *R. anatipestifer* CH-2 adding His tag, Kan^R^	This study
pET30a::*cas1^*E*149*A*^*	pET30a carrying *cas1^*E*149*A*^* adding His tag, Kan^R^	This study
pET30a::*cas1^*H*206*A*^*	pET30a carrying *cas1^*H*206*A*^* adding His tag, Kan^R^	This study
pET30a::*cas1^*E*221*A*^*	pET30a carrying *cas1^*E*221*A*^* adding His tag, Kan^R^	This study
pET30a::*cas1-cas2*	pET30a carrying *cas1-cas2* from *R. anatipestifer* CH-2 adding His tag and Flag tag, Kan^R^	This study
pLMF03	B739_0921 promoter, *ori* ColE1, *ori* pRA0726, Amp^R^, Cfx^R^	Liu M. et al., 2016

**Table 2:** Primers used in this study.

**Primer**	**Sequence (5′-3′)**	**Purpose**	**Source or reference**
P001-Δcas1-up-F	ATAAAAATCCGATATGGCTCAACAAAGAAAAAGG	RA-CH-2 Δ*cas1*, Δ*cas1-cas2*	This study
P002-Δcas1-up-R	TGTATTCACGAAAATCCATTATTTTTAATATTCTCC		This study
P003-Δcas1-spc-F	AATAATGGATTTTCGTGAATACATGTTATAATAACT		This study
P004-Δcas1-spc-R	TTAAACTTCATGCTTACCAATTAGAATGAATA	RA-CH-2 Δ*cas1*	This study
P005-Δcas1-down-F	CTAATTGGTAAGCATGAAGTTTAATCGTTTTAATG		This study
P006-Δcas1-down-R	CACTCGCAATCTGCTGTAAATCGTTTTG		This study
P007-Δcas2-up-F	CCTTTGTATGGGCACTCCGAATAT	RA-CH-2 Δ*cas2*	This study
P008-Δcas2-up-R	GTATTCACGAATTAAACTTCATAAAACTCAGGC		This study
P009-Δcas2-spc-F	TGAAGTTTAATTCGTGAATACATGTTATAATAACTA		This study
P010-Δcas2-spc-R	CTTTTTCTATTCGCTTACCAATTAGAATGAATAT	RA-CH-2 Δ*cas2*, Δ*cas1-cas2*	This study
P011-Δcas2-down-F	TAATTGGTAAGCGAATAGAAAAAGACTTTACATC		This study
P012-Δcas2-down-R	GCAACATAGACGAGATTGAGATATGACAACAC		This study
P013-Δcas1-cas2-Ident-F	AAAGATTGAAAAGGTGGTGG	Identification for all the deletion mutant strains	This study
P014-Δcas1-cas2-Ident-R	CGCAACATTTTACACGAAG		This study
P015-cas1-exp-F	CGGAATTCATATGCATCATCATCATCATCACATGCTTT ACCGTTCTATCTAT	pET30a::*cas1*, pET30a::*cas1^*E*149*A*^*, pET30a::*cas1^*H*206*A*^*, pET30a::*cas1^*E*221*A*^*, pET30a::*cas1-cas2*	This study
P016-cas1-exp-R	CCGCTCGAGTTAAACTTCATAAAACTCAGG	pET30a::*cas1*, pET30a::*cas1^*E*149*A*^*, pET30a::*cas1^*H*206*A*^*, pET30a::*cas1^*E*221*A*^*	This study
P017-cas2-exp-R	CCGCTCGAGCTACTTATCGTCGTCATCCTTGTAATC AAAAAGTTCTAATTGC	pET30a::*cas1-cas2*	This study
P018-CRISPR-F	AAACCAACCTATCAGCAA	pGEM::CRISPR	This study
P019-CRISPR-R	AATGGATTTTGGGAGTAT		This study
P020-E149A-R	TGCAATGCCTGCCATATTGGTG	pET30a::*cas1^*E*149*A*^*	This study
P021-E149A-F	CACCAATATGGCAGGCATTGCA		This study
P022-H206A-R	ATTTATTCTTAGCAAAAATCCCCAATACAGGC	pET30a::*cas1^*H*206*A*^*	This study
P023-H206A-F	CCTGTATTGGGGATTTTTGCTAAGAATAAAT		This study
P024-E221A-R	ACGGTAAGGTGCCATTAAATCGTCT	pET30a::*cas1^*E*221*A*^*	This study
P025-E221A-F	ACGATTTAATGGCACCTTACCGT		This study

### Construction of deletion mutant strains

To generate deletion mutant strains, ~600-bp-long flanking sequences upstream and downstream of RA-CH-2 *cas1* or *cas2* were amplified by PCR using the primers described in Table 2. The sequence containing the SpcR cassette was amplified from the plasmid pAM238 (Fournier et al., 2011) and used as a selectable marker. The three PCR fragments (upstream, downstream, and SpcR cassette) were amplified using the overlap PCR method (Xiong et al., 2006). The mutant strains Δ*cas1*, Δ*cas2*, and Δ*cas1-cas2* were constructed by homologous recombination using the natural transformation method described by Liu et al. (2017b). Briefly, the PCR fragment (up-Spc-down) was mixed with 300 μl of RA-CH-2 (OD_600_ = 1). After incubation for 1 h at 37°C, the mixture was spread onto plates supplemented with Cfx and incubated overnight at 37°C until the appearance of colonies for PCR screening. Using the same method, the shuttle plasmid pLMF03 (Liu M. et al., 2016) was introduced into RA strains by natural transformation.

### Construction of expression plasmids

To prepare expression plasmids, the *cas1* gene of RA-CH-2 was amplified by PCR, digested by *NdeI*/*XhoI* and cloned into a similarly digested pET30a plasmid. Site-directed mutagenesis was performed by overlap PCR (Xiong et al., 2006) to substitute the residue E149, H206, or E221 of Cas1 with an alanine (Cas1^E149A^, Cas1^H206A^, or Cas1^E221A^). The *cas1*-mutant DNA fragment was obtained by two rounds of PCR and similarly cloned into pET30a to produce pET30a::*cas1*^*E*149*A*^, pET30a::*cas1*^*H*206*A*^, and pET30a::*cas1*^*E*221*A*^. The *cas1-cas2* sequence was amplified by PCR from RA-CH-2 genomic DNA and cloned into the pET30a plasmid. *E. coli* BL21 (DE3) cells were transformed with the above plasmids using the calcium chloride method as described by Sambrook and Russell (2001).

### Detection of spacer acquisition using PCR

The strains carrying the shuttle plasmid were grown for 18 h at 37°C in TSB containing 1 μg/ml Cfx. The cells were then collected and frozen at −80°C in 20% (v/v) glycerol to be used as inocula in subsequent experiments. Each strain was inoculated into 5 ml of TSB without antibiotics and grown for 16 h with shaking (defined as one growth cycle). This process was performed for 20 growth cycles. At the same time, a volume of the culture (from cycles 1, 5, 10, 15, and 20) equivalent to 0.3 ml of the culture with an OD_600_ of 1.0 was pelleted by centrifugation, resuspended in 40 μl of water, boiled at 98°C for 10 min and centrifuged at 12,000 rpm for 1 min. The supernatant was used as the template for PCR amplification of the CRISPR1 locus from RA-CH-2. PCR products were analyzed on 2% TAE agarose gels stained with GoldView (SBS Genetech, China) to detect CRISPR1 array expansion. In addition, each single colony on antibiotic-free TSA plates was diluted in 20 μl of water and used as a PCR template. The PCR products of colonies with expanded arrays were submitted for sequencing.

### *In vivo* plasmid loss assays

Serial dilutions of the indicated culture at growth cycles 1, 5, 10, 15, or 20 were spread on TSA with or without 1 μg/ml Cfx, and the plates were incubated for 24 h at 37°C until colonies were visible. The plasmid loss rate was calculated by the following equation: 1-(the number of CFUs on the TSA-Cfx plate)/(the number of CFUs on the TSA plate). Experiments were performed in triplicate.

### Analysis of new spacers and the protospacer adjacent motif (PAM)

Sequences of the expanded CRISPR1 arrays were analyzed using the CRISPRFinder program (http://crispr.i2bc.paris-saclay.fr/) (Grissa et al., 2007), and new spacer sequences were compiled and aligned to the target plasmid or genome sequence using SnapGene software (version 2.3.2, from GSL Biotech; available at snapgene.com) to determine the protospacer location and target strand. The protospacers with 10-bp-long flanking sequences were aligned to identify PAMs by using WebLogo (http://weblogo.berkeley.edu/logo.cgi/) (Crooks et al., 2004).

### Expression and purification of the *R. anatipestifer* cas1 and cas1 mutant proteins

Strains *E. coli* BL21 (DE3) pET30a::*cas1, E. coli* BL21 (DE3) pET30a::*cas1*^*E*149*A*^, *E. coli* BL21 (DE3) pET30a::*cas1*^*H*206*A*^, and *E. coli* BL21 (DE3) pET30a::*cas1*^*E*221*A*^ were grown overnight in LB medium containing 50 μg/ml Kan. LB broth containing Kan was inoculated to an OD_600_ of 0.05 with the overnight culture, and the cells were grown at 37°C with shaking until the culture density reached an OD_600_ of 0.5. Expression was induced with 0.4 mM (final concentration) IPTG, and the cells were grown for an additional 6 h at 37°C. The induced cultures were harvested, and the cells were resuspended in 50 ml of buffer (pH 8.0, 300 mM NaCl, 50 mM NaH_2_PO_4_, 5 mM imidazole) and sonicated. The supernatant of cell lysates was collected by centrifugation and filtered through a 0.45-μm Millex syringe filter unit (Millipore, USA). The filtrate was subjected to nickel-chelate affinity chromatography (Ni-NTA agarose, Bio-Rad, USA) by following the manufacturer's instructions. Elution of the target protein was performed by competition with increasing concentrations of imidazole. Products from the course of purification were collected and measured by SDS-PAGE, and the protein concentration was determined using the Pierce BSA Protein Assay Kit (Pierce Biotechnology, USA). The purified target protein was dialyzed in 50 mM NaH_2_PO_4_ buffer (pH 8.0).

### Nuclease activity assays

The CRISPR1 sequence from RA-CH-2, including the leader region, 16 repeats and 15 spacers, was cloned into the pGEM-T vector (Promega, USA), serving as the circular double-stranded DNA (dsDNA) substrate. Meanwhile, the pGEM::CRISPR vector was linearized with *Sac*I (4.4 kb), serving as the linear dsDNA substrate. Circular or linear dsDNA substrates (0.15 μg) were incubated with the purified recombinant RA Cas1 protein or Cas1 mutants (1 μg) at 25°C for 90 min in the presence of 0.1 M KCl and 0.02 M HEPES (pH 7.5) and with either no metal ion or 2.5 mM manganese (Mn^2+^); deoxyribonuclease I (DNase I, 1 mg/ml) was used as a positive control for the nuclease reaction. The reaction products were extracted with phenol and analyzed by 1.5% agarose gel electrophoresis as described for the *Pseudomonas aeruginosa* Cas1 nuclease assay (Wiedenheft et al., 2009).

### Preparation of polyclonal antibody

Five hundred microliters of an emulsion containing purified RA Cas1 (0.5 mg) and Freund's adjuvant (250 μl) was inoculated three times (at 1-week intervals) into 2-month-old rabbits (1.5 kg/rabbit). One week after the third immunization, blood samples were collected and centrifuged twice (5,000 rpm for 10 min) to obtain serum, which was then stored at −20°C. Before the serum was used, non-specific antibodies were removed by incubating the immune serum with *E. coli* cell extract for 1 h at 4°C, followed by centrifugation for 10 min at 8,000 rpm. The supernatant was then used as a polyclonal antibody.

### Western blotting assays

Protein bands on SDS-PAGE gels were transferred to a polyvinylidene fluoride membrane (PVDF membrane, Millipore). The membrane was blocked with 5% skim milk and incubated with the rabbit polyclonal antibody against RA Cas1 protein or the FLAG-tag and then with goat antirabbit IgG-HRP (ZSGB-BIO, China). Protein bands on the membrane were visualized by the HRP-DAB Chromogenic Substrate Kit (Tiangen Biotech, China).

### Ni-NTA pull-down assays

The Cas1-His and Cas2-FLAG proteins were expressed from pET30a::*cas1-cas2* in *E. coli* BL21 (DE3). Cell lysates of induced cultures (~10^8^ CFU) were incubated with 50 μl of Ni-NTA agarose (Bio-Rad) that was pre-equilibrated with binding buffer (50 mM NaH_2_PO_4_, 300 mM NaCl, pH 8.0) for 16 h at 4°C. After incubation, the resin was washed four times with binding buffer containing 10 mM imidazole to remove non-specifically bound proteins. The resin was then resuspended in 30 μl of SDS loading buffer, heated to 100°C for 5 min and used for Western blot analysis using the anti-FLAG (Beyotime, China) as a primary antibody.

### Immunoprecipitation assays

Cell lysates with pET30a::*cas1-cas2* (~10^8^ CFU) were prepared as described above and incubated with rabbit anti-Cas1 polyclonal antibody overnight at 4°C with gentle shaking, followed by the addition of protein A+G agarose (Beyotime) for 2 h at 4°C. The samples above were washed five times with PBS, separated by SDS-PAGE and detected by Western blotting using anti-FLAG (Beyotime) or anti-Cas1 as primary antibodies.

### Ethics statement

Animal experiments were performed in strict compliance with the guidelines of the National Institutes of Health, and all procedures were approved by the Institutional Animal Care and Use Committee of Sichuan Agricultural University in Sichuan, China (Protocol Permit Number: XF2014-18).

### Statistical analysis

Statistical analysis was performed using GraphPad Prism 6 software. Statistical significance of the data was ascertained using Student's *t*-test. *P*-values < 0.05 were considered statistically significant.

## Results

### *R. anatipestifer* CH-2 was able to acquire a new spacer after the introduction of an exogenous plasmid

**Figure 2:**
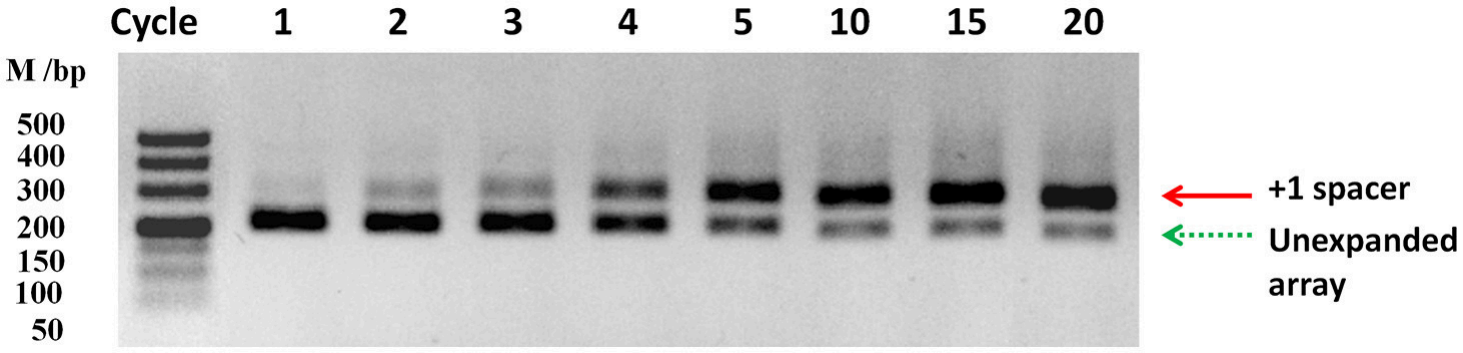
Spacer acquisition assays. The 5′ end of the CRISPR1 locus in RA-CH-2 population (harboring the shuttle plasmid pLMF03) was monitored by PCR amplification using the primers indicated in Figure 1 for the culture at the indicated growth cycle. Each growth cycle represents a 3:1,000 dilution of a previous 16-h culture grown for an additional 16 h in TSB without antibiotics. The red solid arrow represents a new spacer integrated into the CRISPR1 array (~280 bp), while the green dotted arrow represents the unexpanded array (~200 bp). The image is representative of multiple experiments.

The organization of the type II-C CRISPR1-Cas system from RA-CH-2 is shown in Figure 1. The CRISPR adaptation process of the type I-E system (Yosef et al., 2012; Díez-Villaseñor et al., 2013; Arslan et al., 2014; Nuñez et al., 2015) and the type II-A system (Heler et al., 2015; Wei et al., 2015b; Xiao et al., 2017) has been well studied; however, whether the type II-C CRISPR-Cas system is also capable of acquiring new spacers by itself remains unknown. To detect spacer acquisition of RA CRISPR-Cas system, the shuttle plasmid pLMF03 (Liu M. et al., 2016) was introduced into RA-CH-2 using natural transformation (Liu et al., 2017b). In addition, the 5′ end of RA-CH-2 CRISPR1 array was monitored by PCR using the primers shown in Figure 1 and Table 2 for the cultures sampled after the 1st−5th, 10, 15, and 20th growth cycles as described in the Materials and Methods section. As evidenced by the bands shown in Figure 2, two PCR products were amplified from the cultures sampled after two or more growth cycles but not from the culture sampled after one growth cycle. The higher band (~280 bp) was predicted to be the result of one spacer-repeat unit (~80 bp) being added to the CRISPR1 array, while the lower band represented the unexpanded array (Figure 2). Moreover, the higher band gained intensity as the growth cycles increased; in contrast, the lower band became increasingly weaker. This finding suggested that RA-CH-2 was able to acquire new spacers after the introduction of an exogenous plasmid.

### The active process of spacer acquisition contributed to the decreased stability of the shuttle plasmid in *R. anatipestifer* CH-2

**Figure 3:**
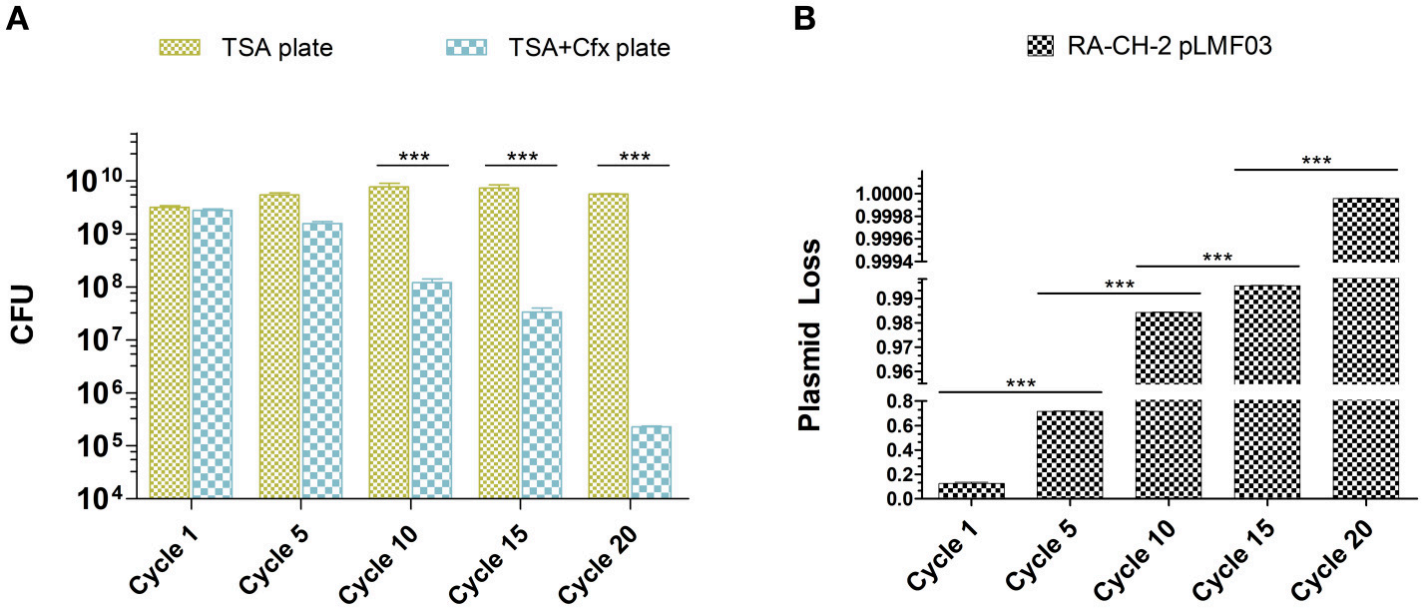
Plasmid loss assays. **(A)** Colony count of the cultures at growth cycles 1, 5, 10, 15, and 20 on TSA plates with or without antibiotics. **(B)** Plasmid loss rates of the cultures. The plasmid loss rate was calculated by the following equation: 1-(the number of CFUs on the TSA-Cfx plate)/(the number of CFUs on the TSA plate). Data were obtained from three independent experiments and analyzed using Student's *t-*test. The asterisks above the line indicate significance between the two indicated groups. ^***^p < 0.001.

Previous studies have shown that CRISPR-Cas system provides resistance against the entry of MGEs by acquiring spacers that match the MGEs (Barrangou et al., 2007; Deveau et al., 2008; Garneau et al., 2010; Sapranauskas et al., 2011). The shuttle plasmid pLMF03 triggered spacer acquisition of RA-CH-2 CRISPR-Cas system (Figure 2), raising the question of whether new spacers contribute to CRISPR/Cas-mediated interference with the plasmid. Thus, plasmid loss assay of the abovementioned cultures (cycles 1, 5, 10, 15, and 20, shown in Figure 2) was carried out by counting the colonies on TSA plates with or without antibiotics (Cfx), as described in the Materials and Methods section. The number of colonies on the TSA plate did not change significantly as the number of growth cycles increased. However, the number of colonies on the TSA plate containing Cfx decreased significantly (Figure 3A). After 20 growth cycles, the number of colonies on the Cfx-containing plate decreased to ~10^5^ CFUs from ~10^9^ CFUs (Figure 3A). Simultaneously, the rate of plasmid loss of the cultures was calculated. As shown in Figure 3B, after five or more growth cycles, more than 70% of the bacteria lost the shuttle plasmid pLMF03 (Figure 3B).

### Cas1 and cas2 of *R. anatipestifer* CH-2 were required for spacer acquisition

**Figure 4:**
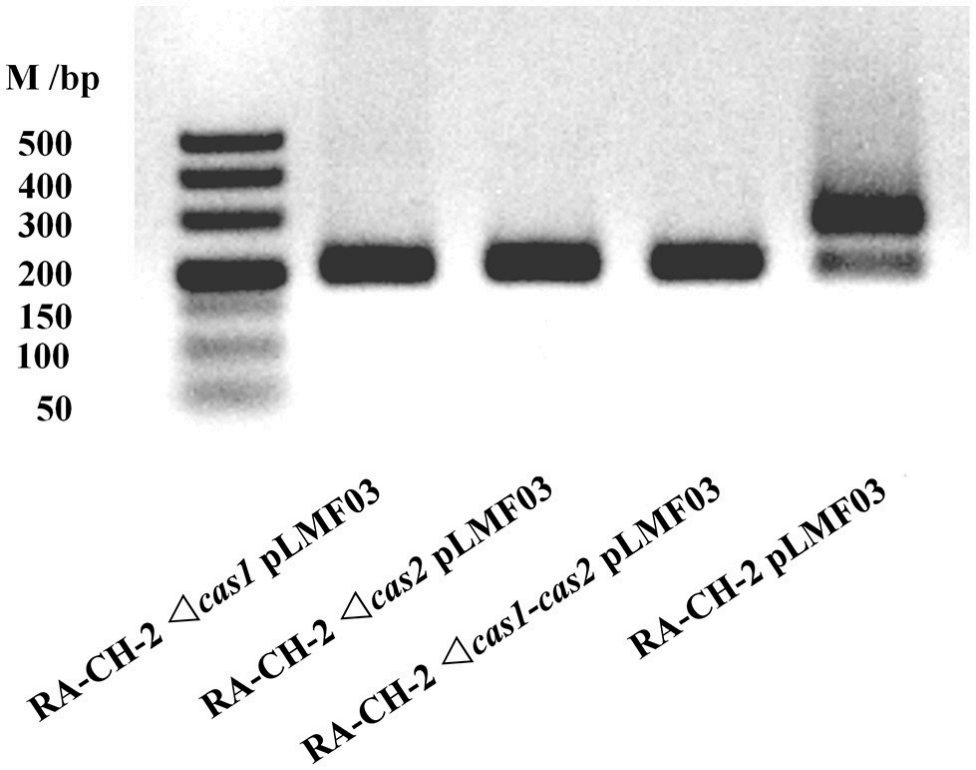
Cas1 and Cas2 were essential for RA-CH-2 spacer acquisition. Cultures of RA-CH-2 strains (wild-type and *cas* gene deletion mutant strains) harboring the shuttle plasmid pLMF03 were grown in TSB without antibiotics for 10 growth cycles. A PCR assay of the cultures was performed with the primers indicated in Figure 1 to detect spacer acquisition. The image is representative of multiple experiments.

Previous studies have indicated that Cas1 and Cas2 participate in the adaptation phase of CRISPR-Cas systems (Yosef et al., 2012; Arslan et al., 2014; Heler et al., 2015; Nuñez et al., 2015; Wei et al., 2015b; Fagerlund et al., 2017; Xiao et al., 2017). To determine the role of RA-CH-2 Cas1 and Cas2, the mutant strains RA-CH-2 Δ*cas1*, RA-CH-2 Δ*cas2*, and RA-CH-2 Δ*cas1-cas2* were constructed as described in the Materials and Methods section. The shuttle plasmid pLMF03 was introduced into the wild-type and mutant strains. These strains were cultured in TSB without antibiotics, and spacer acquisition assay of the cultures through passages for 10 growth cycles was performed using PCR. The expanded CRISPR1 arrays (with increased sizes of PCR products, ~280 bp) were observed in the wild-type strain (Figure 4, Lane 1). However, the PCR products amplified from the mutant strains had sizes corresponding to the unexpanded CRISPR1 array (~200 bp) (Figure 4, Lanes 2–4), indicating the absence of CRISPR expansion in the mutant strains. Spacer acquisition of the *cas1* or *cas2* deletion mutant was rescued by the introduction of the shuttle plasmid expressing both Cas1 and Cas2 (data not shown). These results suggested that Cas1 and Cas2 were required for spacer acquisition of RA*-*CH-2 CRISPR-Cas system.

### Deletion of the *cas1* or *cas2* gene of *R. anatipestifer* CH-2 increased the stability of the shuttle plasmid

**Figure 5:**
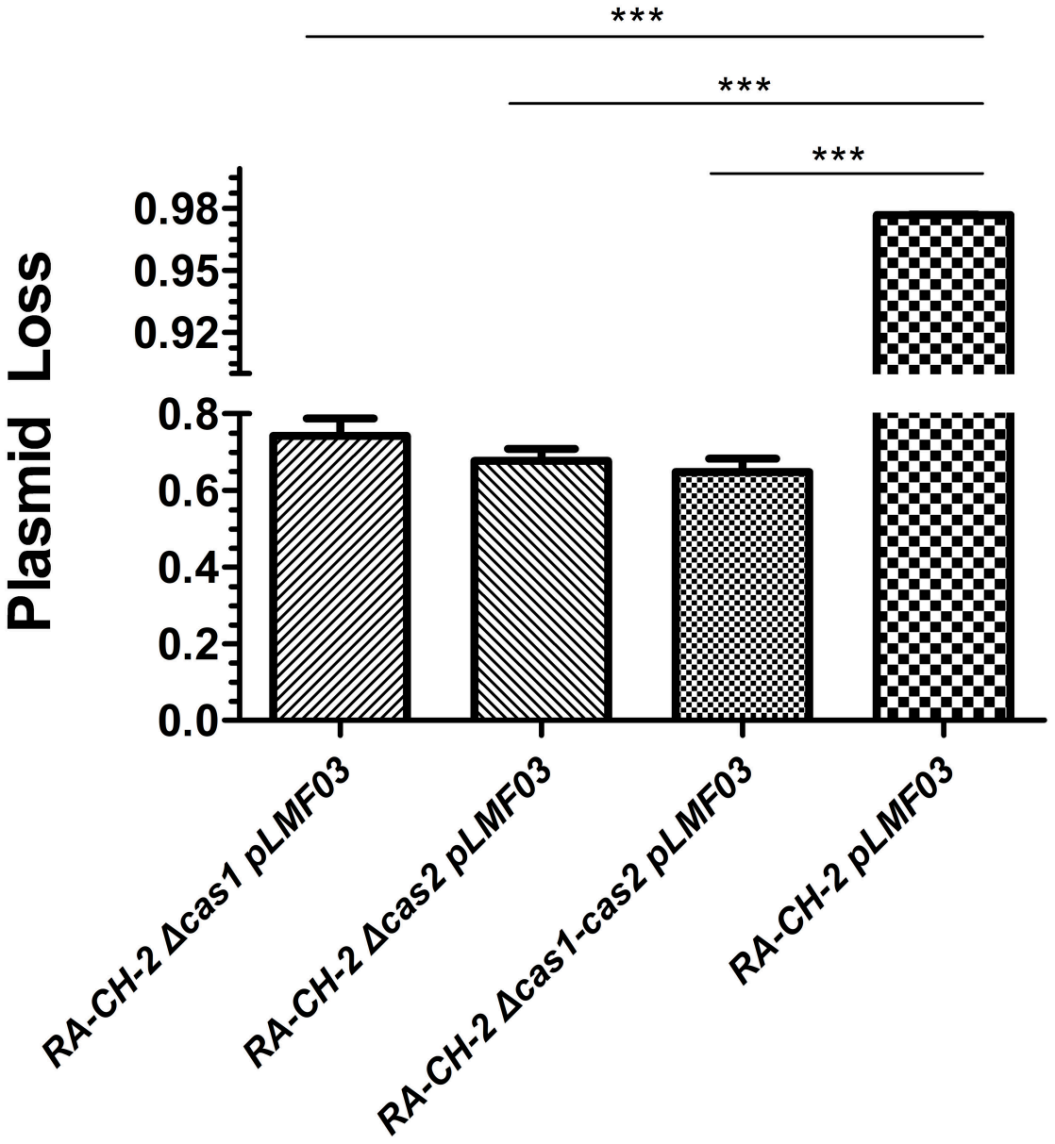
Deletion of RA-CH-2 *cas1* or *cas2* stabilized the shuttle plasmid. Plasmid loss from RA-CH-2 strains (wild-type and *cas* gene deletion mutant strains harboring pLMF03) were analyzed by counting the colonies on plates with or without antibiotics (Cfx). The rate of plasmid loss was calculated, and data obtained from three independent experiments were analyzed using Student's *t*-test. ^***^p < 0.001.

To identify whether inactivating spacer acquisition affects the stability of the shuttle plasmid, plasmid loss assay of the abovementioned cultures (wild-type and mutant strains with pLMF03, as shown in Figure 4) was performed as described previously. The plasmid loss rate of the mutant strains was significantly lower than that of the wild-type strain (Figure 5). This result revealed that deletion of RA-CH-2 *cas1* or *cas2* gene largely eliminated spacer acquisition of the CRISPR-Cas system and subsequently stabilized the shuttle plasmid.

### The newly inserted spacers were derived from the shuttle plasmid

**Figure 6:**
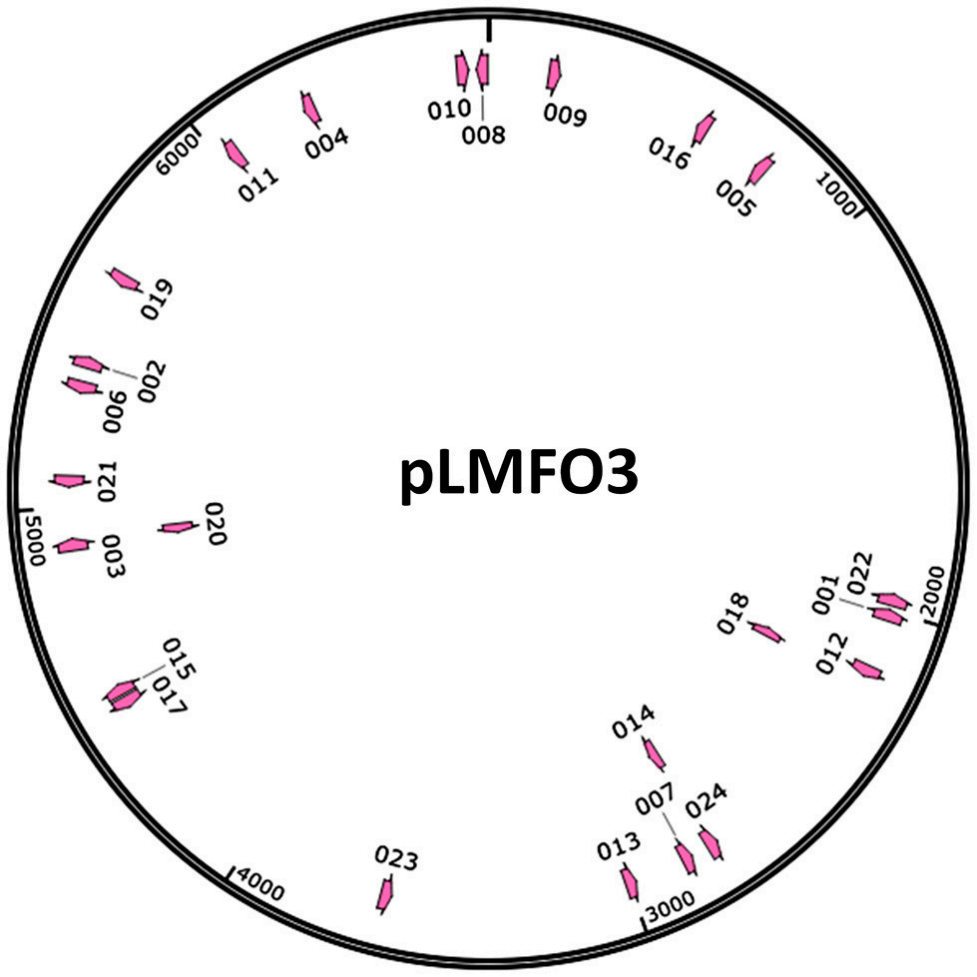
Distribution of the protospacers. The protospacers were numbered based on the sequenced spacers (as listed in Table 3) and are indicated on the pLMF03 plasmid map according to their positions and orientations (pink arrows). Arrows pointing in the clockwise direction represent protospacers matching the plus strand of the pLMF03 vector, and those pointing in the counterclockwise direction represent protospacers matching the minus strand.

**Figure 7:**
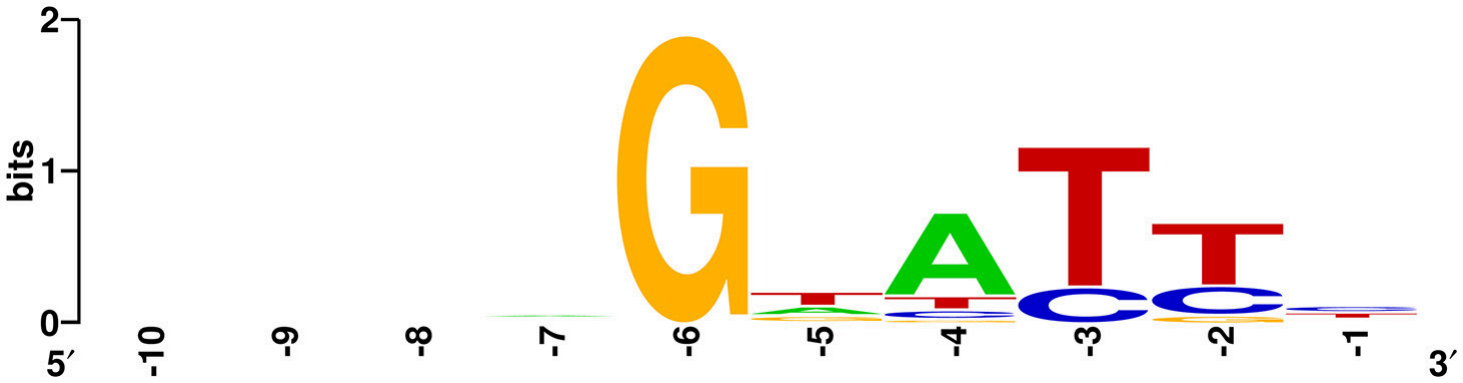
Prediction of the PAM using WebLogo. The flanking sequences upstream of the protospacers (10 nt) were aligned using WebLogo. The first nucleotide (at position −1) was adjacent to the 5′ end of the protospacer. The sizes of the letters indicate the relative frequency of the corresponding base at that position, and the conserved PAM was predicted to be 5′-GWATTN-3′ (W represents A or T).

To further characterize the newly acquired spacers, expanded CRISPR1 arrays of 34 colonies were sequenced and analyzed. Sequencing data showed that the new spacer-repeat units were inserted into RA*-*CH-2 CRISPR1 array at the leader-proximal terminus (between *cas2* and the first repeat, shown in Figure 1). Twenty-four unique spacers were obtained and are listed in Table 3. The length of most of the spacers was 30 bp, which is consistent with the observed spacer length in RA-CH-2 CRISPR1 array, with the exception of one spacer that was 31 bp in length. All spacers were derived from pLMF03 via sequence analysis. The positions and orientations of the protospacers are indicated on the pLMF03 plasmid map (Figure 6). Previous studies have shown that PAM plays an important role in the selection of the protospacer (Mojica et al., 2009; Wang et al., 2015; Leenay and Beisel, 2017). To identify the PAM sequence, 10-bp-long flanking sequences upstream or downstream of the protospacers were aligned by WebLogo. The observed PAMs were located immediately upstream of each protospacer, and the conserved motif was predicted as 5′-GWATTN-3′ (W represents A or T) (Figure 7 and Table 3). The first nucleotide of the PAMs, G, was significantly more conserved than the other bases.

**Table 3:** Analysis of new spacers.

**Number**	**Spacer sequence**	**Spacer length (nt)**	**Source^a^**	**Location^b^**	**Strand^c^**	**PAM^d^ (5′−3′)**
001	GATATGTTGTCGCTTAGTGTCGTTATGTGT	30	Plasmid	1997.2026	Minus	GTATTT
002	ACGCCGGGCAAGAGCAACTCGGTCGCCGCA	30	Plasmid	5363.5392	Plus	GTATTG
003	CACCGTCATCACCGAAACGCGCGAGACGAAA	31	Plasmid	4892.4922	Plus	GGTTTT
004	GCGCTCTGCTGAAGCCAGTTACCTTCGGAA	30	Plasmid	6248.6277	Minus	GTATCT
005	TAAAAGTTCATTATTTTTATATAACTTCCT	30	Plasmid	740.769	Minus	GTATTC
006	TAAAAGTGCTCATCATTGGAAAACGTTCTT	30	Plasmid	5297.5326	Minus	GAACTT
007	TGCTGTTTCTGCATCTGCTGTACTTGGCTC	30	Plasmid	2824.2853	Minus	GTATCA
008	CCATAGGCTCCGCCCCCCTGACGAGCATCA	30	Plasmid	6703.6732	Minus	GTTTTT
009	AGCGAGTCAGTGAGCGAGGAAGCGGAAGAG	30	Plasmid	156.185	Plus	GAGCGC
010	TTATAGTCCTGTCGGGTTTCGCCACCTCTG	30	Plasmid	6654.6683	Plus	GTATCT
011	CACCTAGATCCTTTTAAATTAAAAATGAAG	30	Plasmid	6022.6051	Minus	GATCTT
012	CCTCATATTGAATACTTCACAGAAGAGGAA	30	Plasmid	2151.2180	Plus	GAATTT
013	CAGCATGGCACAAAACTTTTCCATGCGCAG	30	Plasmid	2981.3010	Minus	GAATCC
014	GAACGTGCCAACGAAAAACTGCAAGACGAG	30	Plasmid	2759.2788	Plus	GAACTG
015	CCGGGTACCGAGCTCGAATTCACTGGCCGT	30	Plasmid	4500.4529	Plus	GGATCC
016	TAAAAGTTCACGAATGAAAAAAAACAAACA	30	Plasmid	561.590	Minus	GTATTC
017	CCAAAATCAGTTCTTTAGCGATTACTAATT	30	Plasmid	4464.4493	Minus	GGATCC
018	TGTGATCCTTTTTTTAAACTCTTGGTATTC	30	Plasmid	2178.2207	Minus	GTACTC
019	ATTCAGCTCCGGTTCCCAACGATCAAGGCG	30	Plasmid	5590.5619	minus	GGCTTC
020	CGAGGCCCTTTCGTCTCGCGCGTTTCGGTG	30	Plasmid	4901.4930	Minus	GTATCA
021	ATCAGGGTTATTGTCTCATGAGCGGATACA	30	Plasmid	5054.5083	Minus	GCATTT
022	TCCGTTCGCTGACGCCAAAAAGCTCTGCGA	30	Plasmid	1957.1986	Minus	GGATTG
023	AAAACAGGTGTAGATAGGATAGCAGCTCCA	30	Plasmid	3616.3645	Minus	GAATGC
024	CAGTTTTTCGTTGGCACGTTCCAGTTCTTT	30	Plasmid	2750.2779	Minus	GTCTTG

a Protospacers are derived from the pLMF03 plasmid.

b Based on plasmid sequence numbering in KU997673.1.

c *Protospacer matches the plus or minus strand of the source*.

d *Protospacer adjacent motifs are highlighted in red*.

### The interaction of cas1 and cas2 of *R. anatipestifer* CH-2 *in vitro*

**Figure 8:**
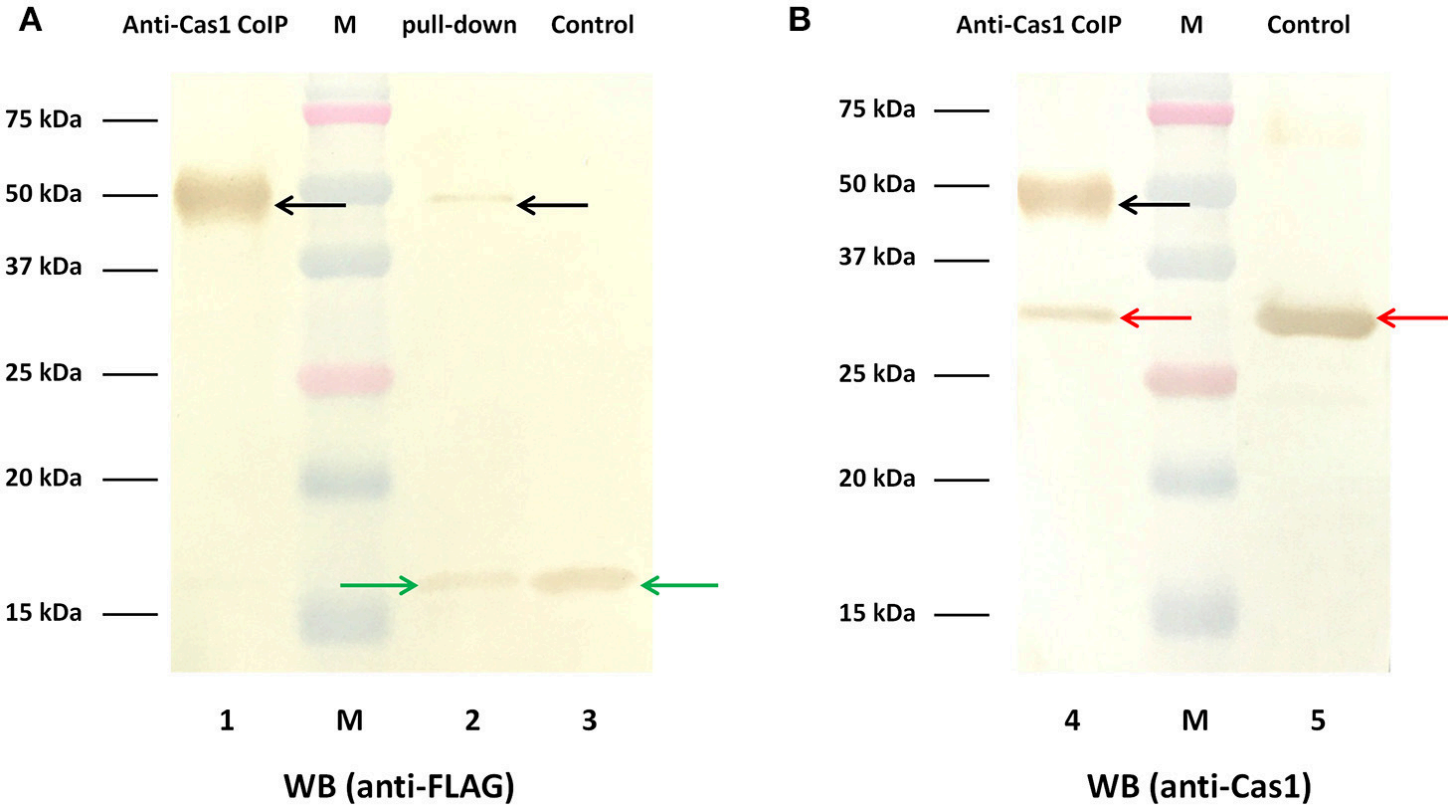
Interaction of RA Cas1 and Cas2 *in vitro*. **(A,B)** Western blotting using anti-FLAG and anti-Cas1 antibodies, respectively. Lanes 1 and 4, the samples immunoprecipitated by anti-Cas1. Lane 2, the pull-down sample obtained by using Ni-NTA agarose. Lanes 3 and 5, the untreated control samples (cell lysates before assay).

The above results show that deletion of the *cas1* or *cas2* gene largely eliminated spacer acquisition. Correspondingly, the plasmid loss rate of the *cas1* or *cas2* deletion mutant strain was lower than that of the wild-type strain. It has been shown that in CRISPR-Cas systems from *E. coli* (Nuñez et al., 2014, 2015; Wang et al., 2015; Künne et al., 2016; Yoganand et al., 2017), *Pectobacterium atrosepticum* (Fagerlund et al., 2017), *Streptococcus pyogenes* (Heler et al., 2015), and *Enterococcus faecalis* (Xiao et al., 2017), interaction of Cas1 and Cas2 is required for these proteins to function in spacer acquisition. Thus, we asked whether Cas1 interacts with Cas2 to function in RA-CH-2 CRISPR-Cas system in the same pathway. To test the interaction of Cas1 and Cas2 *in vitro*, expression of Cas1-His and Cas2-FLAG fusion proteins was induced in BL21 (DE3) cells, and cell lysates were used to conduct Ni-NTA pull-down assays and immunoprecipitation assays. The pull-down sample from the Ni-NTA agarose assay exhibited two protein bands of different sizes by Western blotting using the anti-FLAG antibody (Figure 8, Lane 2). The protein with a molecular weight of ~50 kDa was predicted to be the complex of Cas1-His (35.5 kDa) and Cas2-FLAG (14.4 kDa), and the other protein, with a molecular weight of ~15 kDa, was the Cas2-FLAG fusion protein and was the same size as that observed in the untreated control sample (cell lysates before assay, Figure 8, Lane 3). The sample immunoprecipitated by the anti-Cas1 polyclonal antibody also presented the band corresponding to the His-Cas1-Cas2-FLAG complex (with a molecular weight of ~50 kDa) by Western blotting using either anti-FLAG or anti-Cas1 antibody (Figure 8, Lane 1 and 4), which was consistent with the pull-down assay. These results revealed that RA Cas1 and Cas2 interacted directly.

### *R. anatipestifer* CH-2 cas1 exhibited DNA nuclease activity

**Figure 9:**
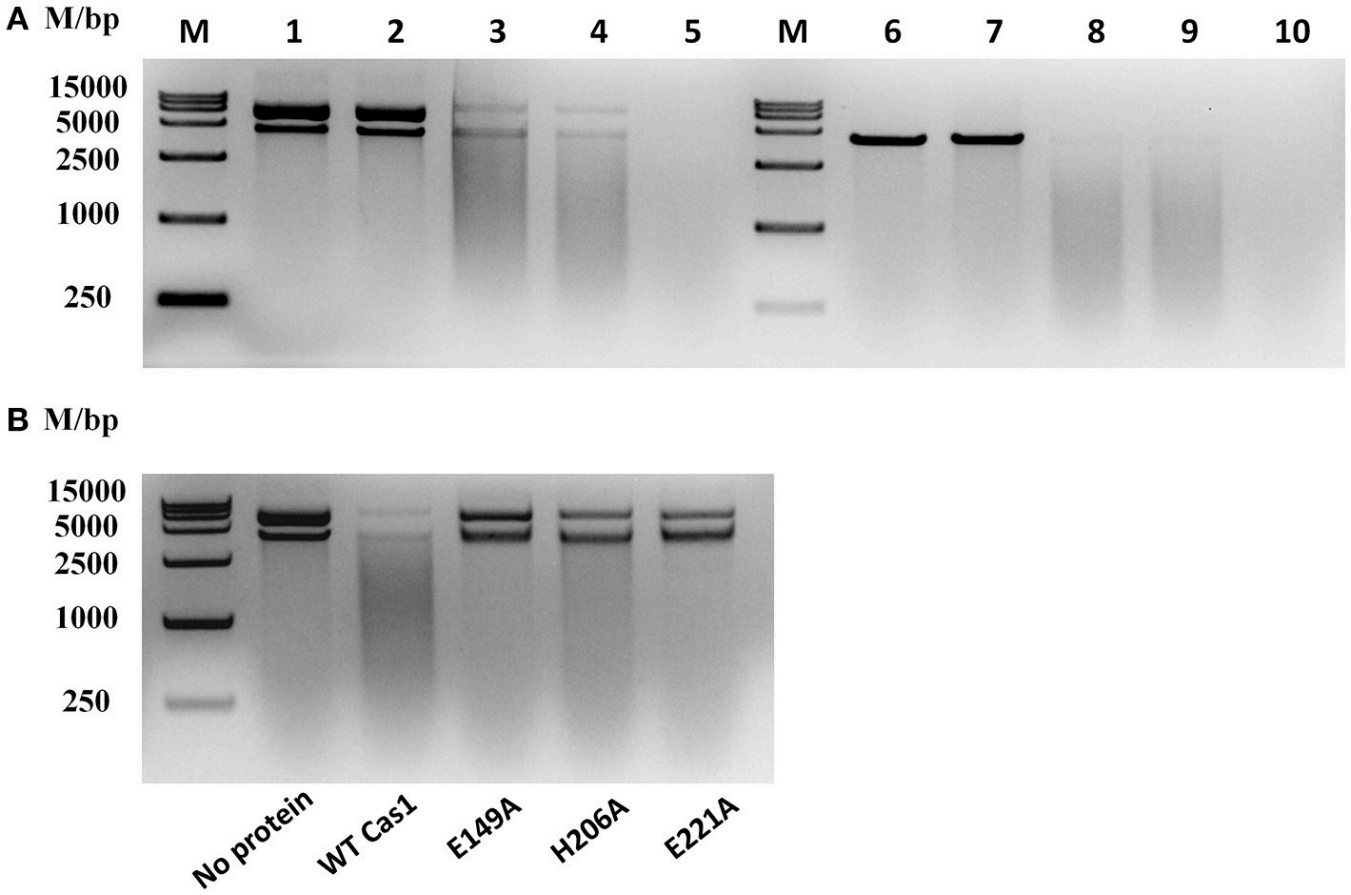
Nuclease activity assays for RA Cas1. **(A)** The recombinant RA Cas1 protein degraded circular and linear dsDNA. Lanes 1–5, circular dsDNA substrate. Lanes 6–10, linear dsDNA substrate. Lanes 1 and 6, no protein (no Cas1). Lanes 2 and 7, no metal ion (no Mn^2+^). Lanes 3, 4, 8, and 9, nuclease reactions of the recombinant Cas1 protein in the presence of Mn^2+^; Lanes 4 and 9, reaction products extracted with phenol. Lanes 5 and 10, deoxyribonuclease I (1 mg/ml DNase I) used for the nuclease reaction as a positive control. **(B)** Nuclease activity assays for Cas1 mutants; reaction products were extracted with phenol before electrophoresis. All nuclease reaction products were analyzed by 1.5% agarose gel electrophoresis.

To determine whether RA-CH-2 Cas1 exhibits DNA nuclease activity, DNase activity of the recombinant Cas1 protein was explored by adding this protein to circular or linear dsDNA substrates in the presence of 2.5 mM Mn^2+^ (or no metal ion), 0.1 M KCl, and 0.02 M HEPES (pH 7.5) at 25°C for 90 min. The electrophoretic profiles show that both the circular and linear dsDNA substrates were degraded by recombinant Cas1 to non-specific smear products in the presence of Mn^2+^ (Figure 9A, Lanes 3, 4, 8, and 9). However, the nuclease reactions without the Cas1 protein or Mn^2+^ abolished the cleavage of the dsDNA substrates (Figure 9A, Lanes 1, 2, 6, and 7), suggesting that Mn^2+^ assisted RA Cas1 recombinant protein in the cleavage of dsDNA.

By sequence analysis using the UniProt Knowledgebase (UniProtKB), three conserved residues of RA-CH-2 Cas1, namely, E149, H206, and E221, were predicted as functional sites for DNA binding and nuclease activity. To determine the roles of these residues in the nuclease activity of RA Cas1, Cas1 mutant proteins (Cas1^E149A^, Cas1^H206A^, and Cas1^E221A^, generated by site-directed mutagenesis) were expressed and purified for nuclease activity assays. In comparison with wild-type Cas1, the Cas1 mutants were unable to completely degrade dsDNA into smaller fragments under the conditions used in this study (Figure 9B). The above results suggested that RA Cas1 was a metal-dependent nuclease and was able to degrade circular and linear dsDNA. In addition, the results indicated that residues E149, H206, and E221 were the active sites of RA Cas1 nuclease.

## Discussion

Subtype II-C CRISPR-Cas loci universally contain a trans-activating crRNA (tracrRNA), three *cas* genes (*cas9, cas1*, and *cas2*), a leader sequence (A/T-rich), and CRISPR (repeats and spacers) (Chylinski et al., 2014). In addition to containing these elements, subtype II-A and II-B loci contain Csn2 or Cas4, and these proteins have been found to be essential components for CRISPR adaptation (Li et al., 2014; Heler et al., 2015; Wei et al., 2015b). Subtype II-C CRISPR-Cas systems are prevalent in pathogens and commensals and are the most common subtypes among type II systems; however, these subtypes are the least well studied (Chylinski et al., 2014). Previously, the only report of type II-C adaptation was from *Campylobacter jejuni*, and the *C. jejuni* adaptation was unusual (Hooton and Connerton, 2014).

RA, the organism used in this study, is a pathogenic bacterium with high morbidity and mortality that primarily infects domestic ducks (Ruiz and Sandhu, 2013). Similar to *C. jejuni*, new spacer-repeat units from RA were inserted into the CRISPR array at the leader-proximal terminus (between *cas2* and the first repeat, as shown in Figure 1), suggesting that spacer acquisition of type II-C system is generally directional (more data on the adaptation of type II-C systems from other organisms are needed to support this hypothesis). However, the adaptation of the *C. jejuni* CRISPR-Cas system requires a Cas4-like protein from the *Campylobacter* bacteriophage to activate spacer acquisition, and all the new spacers observed were derived from *C. jejuni* chromosomal DNA (Hooton and Connerton, 2014), which was quite different from the adaptation observed for RA CRISPR-Cas system.

The type II-C CRISPR-Cas system from RA-CH-2 was extremely active and was able to acquire new spacers after the introduction of an exogenous plasmid (containing no *cas* gene) on its own. All new spacers sequenced from RA-CH-2 were preferentially selected from the extrachromosomal DNA (the shuttle plasmid pLMF03), identical to *E. coli* and *Streptococcus thermophilus*. However, the adaptation of *E. coli* type I-E system (Yosef et al., 2012; Díez-Villaseñor et al., 2013) and *S. thermophilus* type II-A system (Wei et al., 2015b) was triggered by a plasmid expressing Cas1 and Cas2 (or Csn2) but not by an empty plasmid. One possibility is that the expression level of Cas1 and Cas2 was higher in RA-CH-2 but lower in *E. coli* and *S. thermophilus*. Another possibility is that Cas1 and Cas2 of RA-CH-2 were more active than those of *E. coli* and *S. thermophilus*. Interestingly, RNA-Seq data of RA-CH-2 showed that the transcription level of *cas*1 or *cas*2 was not particularly high compared to other genes of RA-CH-2 (our unpublished data). qRT-PCR analysis showed that expression levels of Cas1 and Cas2 in RA-CH-2 carrying the shuttle plasmid pLMF03 were both upregulated slightly, compared with wild type strain (Supplementary Figure 1).

As with the adaptation of *E. coli* and *S. thermophilus* CRISPR-Cas systems or other subtype systems, both Cas1 and Cas2 were required for the adaptation of RA CRISPR-Cas system, and deletion of either *cas1* or *cas2* from RA*-*CH-2 abrogated spacer acquisition, supporting the hypothesis that active adaptation contributes to CRISPR/Cas-mediated interference with the exogenous plasmid. In addition, RA*-*CH-2 Cas1 interacted with Cas2 directly. Moreover, RA Cas1 was able to degrade circular and linear dsDNA in the presence of Mn^2+^, and alanine substitution of residues E149, H206, and E221 abolished the nuclease activity of RA Cas1. It is hypothesized that the nuclease activity of RA Cas1 and the interaction of RA Cas1 with Cas2 are involved in the capture of prespacer substrates and integration of these substrates into the CRISPR array.

The conserved PAM of RA*-*CH-2 CRISPR-Cas system was predicted to be 5′-GWATTN-3′ and was located immediately upstream of the protospacer. The first nucleotide G of the observed PAM of RA-CH-2 system was highly conservative, consistent with the deduced PAM from the putative protospacer (100% similarity) of RA-CH-2 CRISPR1 spacer3. However, the consensus PAMs of other type II systems from *S. pyogenes, S. thermophilus, Streptococcus aureus*, and *Neisseria meningitidis* were located at the 3′ end of the protospacer (Leenay and Beisel, 2017). The location and sequence of the PAMs of those systems were quite different from those of the observed PAM of RA-CH-2 system. This is the first report of the PAM sequence of RA CRISPR-Cas system.

The CRISPR1 array of RA-CH-2 was active for inserting new spacers. However, no CRISPR amplification was observed in the CRISPR2 array (data not shown), probably because there are no *cas* genes flanking the CRISPR2 array of RA-CH-2, or the function of inserting new spacers into CRISPR2 array was lost. This study focused on the naïve adaptation of RA CRISPR-Cas system. It would be interesting to know whether primed adaptation is involved and the requirements in terms of Cas proteins for this process. Previous studies have revealed the role of type II CRISPR-Cas system in bacterial physiology and virulence (Sampson et al., 2013; Serbanescu et al., 2015). In this study, we observed that knockout of *cas1* or/and *cas2* did not have any effect on the growth of RA, and the effects on antibiotic resistance, energy metabolism, physiology, and virulence were not investigated in this study but should be investigated in the future.

The key feature of CRISPR/Cas-mediated immunity against MGEs is the ability to acquire new spacers to continuously update the CRISPR repertory (Jackson et al., 2017). Therefore, adaptation seems to be particularly important in CRISPR-Cas systems. Numerous and varied type II-C CRISPR-Cas systems remain to be explored, especially for their role in adaptation. Here, we provide the first experimental evidence of the naïve adaptation of type II-C CRISPR-Cas system, filling the knowledge gap in this field to a certain extent.

## Author contributions

AC, MW, and YH conceived and designed the project. LH, CL, and YH performed the research. XZ, HY, and DZ participated in the animal experiments. QY, YW, SZ, YL, YY, and LZ contributed to reagents, materials, analysis tools. XXZ, SC, RJ, and YH analyzed the data. MW and YH drafted the manuscript. AC and ML reviewed the manuscript. All authors have read and approved the final version of the manuscript.

### Conflict of interest statement

The authors declare that the research was conducted in the absence of any commercial or financial relationships that could be construed as a potential conflict of interest.
