# Supplementary material for: Cas1 and Cas2 From the Type II-C CRISPR-Cas System of Riemerella anatipestifer Are Required for Spacer Acquisition
Source: Front Cell Infect Microbiol. 2018 Jun 12;8:195. doi: 10.3389/fcimb.2018.00195 (PMC6008519; doi:10.3389/fcimb.2018.00195)
Supplement: Supplementary file 1 [file Image_1.PDF]

Supplementary Material

Cas1 and Cas2 from the type II-C CRISPR-Cas system of *Riemerella anatipestifer* are required for spacer acquisition

Yang He[1,2,3†], Mingshu Wang[1,2,3†], Mafeng Liu[1,2,3], Li Huang[1,2,3], Chaoyue Liu[2,3,4], Xin Zhang[1,2,3], Haibo Yi[1,2,3], Anchun Cheng[1,2,3*], Dekang Zhu[2,3], Qiao Yang[1,2,3], Ying Wu[1,2,3], Xinxin Zhao[1,2,3], Shun Chen[1,2,3], Renyong Jia[1,2,3], Shaqiu Zhang[1,2,3], Yunya Liu[1,2,3], Yanling Yu[1,2,3], Ling Zhang[1,2,3]

*** Correspondence:** Anchun Cheng: chenganchun@vip.163.com.

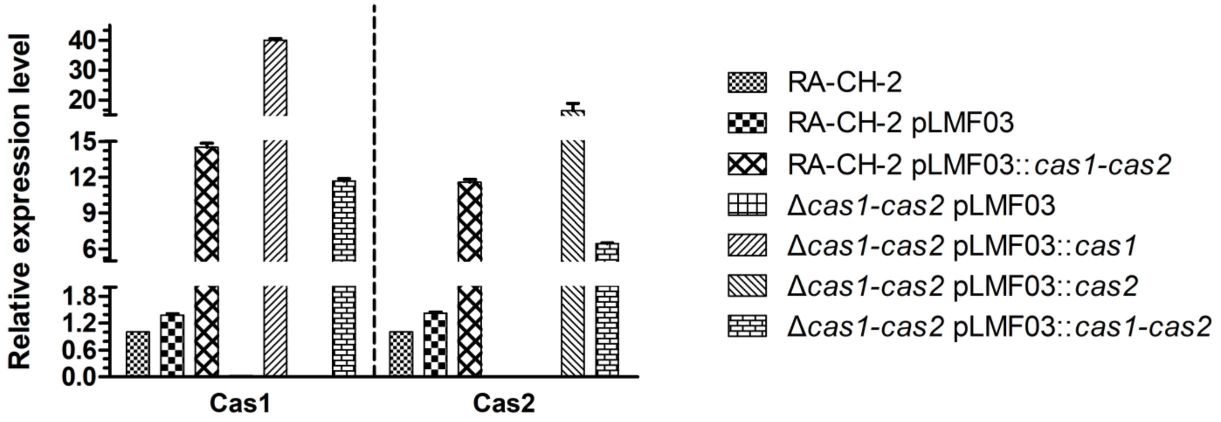

Supplementary Figure 1. qRT-PCR analysis of the relative expression levels of Cas1 and Cas2 in different strains of *R. anatipestifer*. Expression levels of Cas1 and Cas2 in RA-CH-2 carrying the shuttle plasmid pLMF03 were both upregulated slightly (~1.4-fold), compared with wild type strain (RA-CH-2). The mutant strain of *cas1* and *cas2* gene deletion carrying the shuttle plasmid pLMF03 was established as a negative control, and the strain carrying the shuttle plasmid expressing Cas1 or (and) Cas2 as the positive control. *R. anatipestifer* 16S rDNA was used as an internal control. The changes of mRNAs were expressed as fold expression and calculated using the $2^{-\Delta\Delta CT}$ method. Error bars represent the standard deviation of three independent experiments.
